# An Outbreak of Bartonella bacilliformis in an Endemic Andean Community

**DOI:** 10.1371/journal.pone.0150525

**Published:** 2016-03-18

**Authors:** Nuria Sanchez Clemente, Cesar Ugarte-Gil, Nelson Solorzano, Ciro Maguiña, David Moore

**Affiliations:** 1 London School of Hygiene and Tropical Medicine, London, United Kingdom; 2 Instituto de Medicina Tropical Alexander von Humboldt, Universidad Peruana Cayetano, Heredia, Lima, Peru; 3 Hospital San Juan de Dios, Caraz, Peru; University of Malaya, MALAYSIA

## Abstract

**Background:**

Bartonellosis affects small Andean communities in Peru, Colombia and Ecuador. Research in this area has been limited.

**Methods:**

Retrospective review of 191 cases of bartonellosis managed in Caraz District Hospital, Peru, during the last outbreak (2003).

**Results:**

The majority of cases (65%) were 14 years old and younger. There was a peak in acute cases after the rainy season; chronic cases presented more constantly throughout the year. The sensitivity of blood smear against blood culture in acute disease was 25%. The most commonly used treatment for chronic disease was rifampicin; chloramphenicol was used to treat most acute cases. Complications arose in 6.8% and there were no deaths.

**Conclusions:**

Diagnostic and treatment algorithms for acute and chronic bartonellosis have been developed without a strong evidence base. Preparation of ready-to-go operational research protocols for future outbreaks would strengthen the evidence base for diagnostic and treatment strategies and enhance opportunities for control.

## Introduction

**Fig 1:**
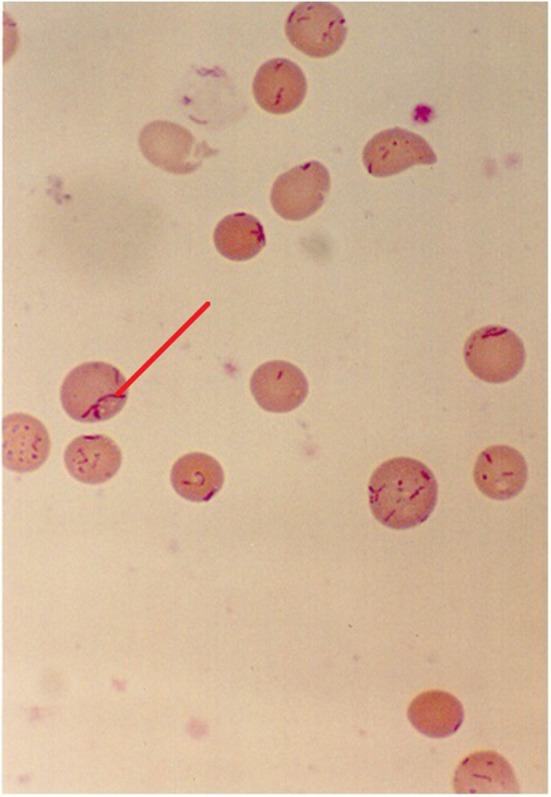
*Bartonella bacilliformis* seen in a blood film with Giemsa staining (1000x magnification). Intra-erythrocytic bacillary and coccoid forms shown.

Bartonellosis, or Carrion’s disease, is a bacterial infection that remains endemic in distinct valleys of the Andean cordillera in Peru, Ecuador, and Colombia. Its causative agent is the gram negative, facultative intracellular, aerobic coccobacillus *Bartonella bacilliformis* (Fig 1) which belongs to the same alpha-proteobacteria group as *Rickettsia*, *Brucella* and others [1].

The disease is widely thought to be transmitted by the sandfly vector *Lutzomyia verrucarum* which has a weak, hopping flight and is intolerant of arid conditions at low altitudes and the cold of higher altitudes [2]. This may explain the focality of the endemic areas, which are characteristically confined to altitudes between 500 to 3200m above sea level [3] and in valleys that are at right-angles to the prevailing wind [1]. The vector has an endophilic, crepuscular feeding habit [4] which means that it preferentially feeds indoors during twilight hours. It has been suggested that El Niño events, which cause a warming in sea temperature every 5–7 years, favourably affect vectors due to a change in climatic conditions. During these periods, it has been shown that the number of cases of bartonellosis in endemic regions increases and in addition, non-endemic areas may experience outbreaks of the disease [5].

The illness manifests as two distinct clinical phases. The first, an acute haemolytic phase, also known as Oroya fever, is characterised by fever and haemolytic symptoms such as pallor and malaise. This phase has been known to have a high mortality of up to 44% to 88% in untreated individuals [6]. The subsequent eruptive phase is known as verruga peruana and classically occurs weeks to months after the acute illness. It is characterised by the appearance of crops of miliary, mular or nodular skin lesions, or verrugas, containing sero-sanguinous fluid which exudes on contact.

The most common complication in the acute phase is secondary infection, most commonly with *Salmonella* species but also with *Toxoplasma*, *Histoplasma* and others [7–8]. Haematological, gastrointestinal [9], cardiovascular [10] and neurological [11] complications have also been well described. In pregnancy, trans-placental transmission has been known to occur, and infection can lead to miscarriage, premature labour and maternal death [1, 12].

Young children are the most affected age group in endemic communities, partly because of a predominantly younger population but also due to the presumed protective immunity that develops with repeated infection [13].

Due to its focal nature and the fact that it affects small, isolated, rural communities, bartonellosis has never received much attention from donors for research and innovation. The latest diagnostic and treatment guidelines [14, 15] are supported by very low evidence studies and expert opinion [16], as large-scale randomised studies have not been carried out. The aim of this study was to characterize the epidemiology, clinical features and approach to management of disease due to *Bartonella bacilliformis* during a recent outbreak in an endemic setting.

## Methods

### Study setting

This study was carried out between July and August 2010 in Caraz, a small Andean town at an elevation of 2300m, 475km northeast of Lima, Peru and with a population of 5000. The town has a 32-bed Ministry of Health regional hospital that offers free diagnostics and treatment for bartonellosis. Nearby villages have small health centres where the poorest residents can receive free healthcare. The hospital’s catchment area includes small settlements up to 12km away and the population it served at the time of the study was around 48626. From 1998 to 2001, members of the Uniformed Services, University of the Health Sciences, Maryland, USA and the Naval Medical Research Centre Detachment, Lima, Peru were stationed in Caraz and granted funding to set up the bartonellosis investigation unit.

### Sample selection

The study focused upon the most recent outbreak in order to look at current manifestations of disease and existing practices in diagnosis and management, and how closely these followed the latest Ministry of Health guidelines at the time, which were issued in 1998. The last outbreak started in 2002 and continued until 2007. The peak year of the outbreak, in which there were the most cases, was 2003 so this was chosen as the study period.

In order to obtain a sample of patients, the bartonellosis investigation unit database was consulted. This database was compiled by chief local epidemiologists and included all cases treated for suspected bartonellosis. Patient information was anonymized prior to analysis and ethical approval was granted to carry out the study both locally by Universidad Peruana Cayetano Heredia’s science and investigation board and by the University of London ethics committee. Cases were defined as patients presenting with fever or those with history of fever in the last 14 days without a focus.

The database was consulted for the year 2003 and 825 cases were found. Due to time constraints and difficulty in accessing case notes, it was not possible to study all 825 cases. It was decided to aim for a sample size of 200. The list of patients was ordered at random and every fourth case was selected. This produced a sample of 206 cases. Of these 206, 66 case notes were missing, so to replace them every third patient was chosen from the list of remaining patients. This produced 50 additional cases, from which 17 case notes were missing, generating a final sample size of 191 cases.

The case notes were scrutinised for information regarding basic demographics, symptoms and signs, diagnostic method, management and complications. Epi Info^TM^ software (CDC) was used to generate an electronic data capture instrument and for the analysis of the data.

## Results

### Participant demographics

**Fig 2:**
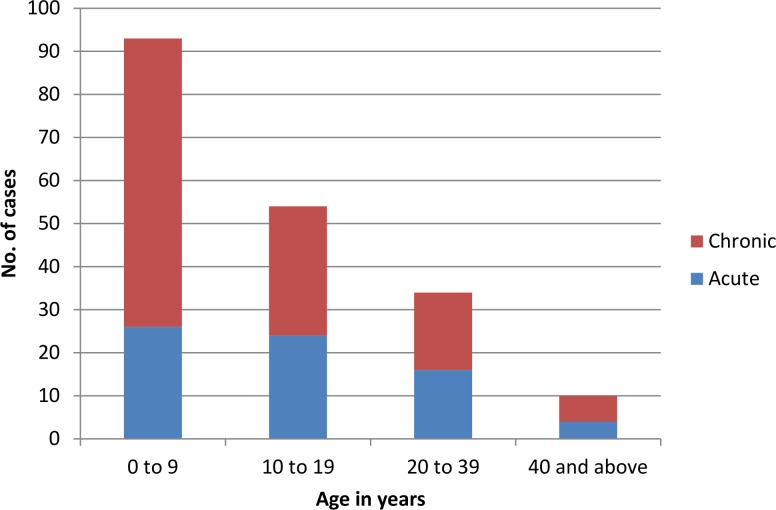
Cases presenting to Caraz Hospital by age group and disease type.

Of the 191 patients reviewed, 52% (100) were male and 48% (91) were female. The mean age of cases was 14.3 years, SD = 14.4 (mean age of males = 13.7, mean age females = 15.3). The median age of patients was 10 years (min = 4 months, max = 82 years, IQR = 15); 63.4% (121) had chronic eruptive disease and 36.6% (70) had acute disease. The distribution of age groups along with disease type is presented in Fig 2. Younger patients tended to present more often with chronic disease than older patients (Fig 2).

**Fig 3:**
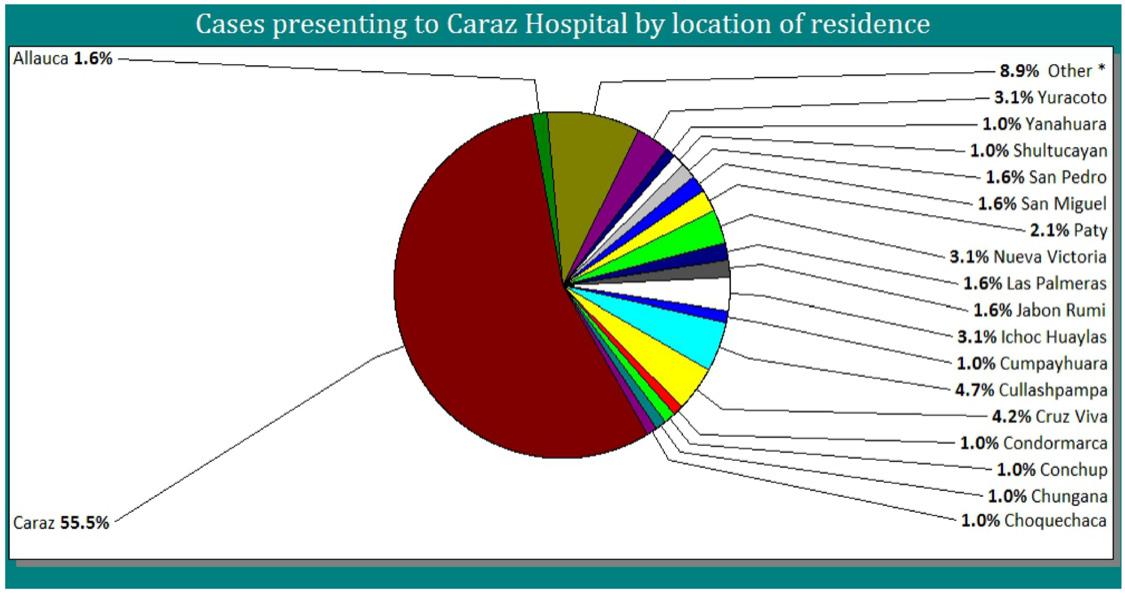
Cases presenting to Caraz Hospital by location of residence.

Just over half (55.5%) of the patients were resident in Caraz, the remainder presented from nearby villages which were 500m to 12km away from Caraz Hospital (Fig 3).

**Fig 4:**
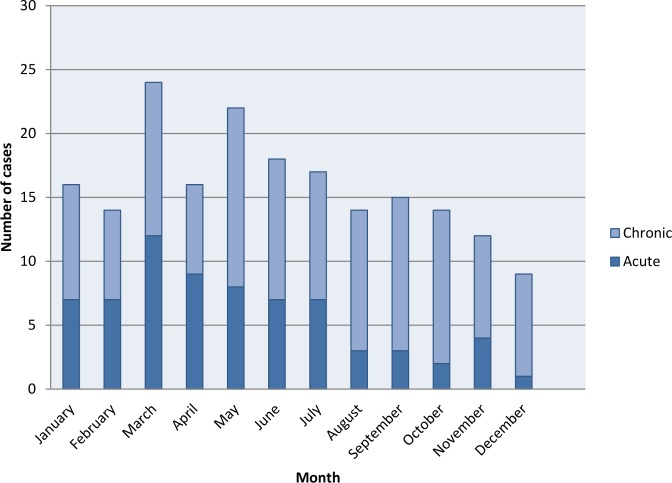
Number of acute and chronic cases presenting per month in Caraz Hospital.

Cases presented evenly across the year but there was a noticeable peak in March and May with a tail off towards the end of the year (Fig 4). The number of acute cases markedly decreased towards the end of the year whereas the number of chronic cases were quite evenly spread with a slight peak in May and September/October.

### Presentation

The predominant features in acute cases were fever, pallor, malaise, joint pain, headache and anorexia (Table 1). However, among chronic cases, patients were often asymptomatic other than their eruptive lesions. The most common symptom associated with the chronic phase was joint pain.

**Table 1:** Clinical features experienced by acute and chronic patients presenting to Caraz Hospital.

Clinical feature	Number (%) of acute patients with feature recorded (n = 70)	Number (%) of chronic patients with feature recorded (n = 121)
fever	56 (80%)	6 (5%)
pallor	39 (55.7%)	3 (2.5%)
malaise	29 (41.4%)	0
joint pain	20 (28.6%)	22 (18.2%)
headache	16 (22.9%)	5 (4.1%)
anorexia	14 (20%)	5 (4.1%)
vomiting	8 (11.4%)	2 (1.7%)
cough	5 (7.1%)	6 (5%)
jaundice	3 (4.3%)	1 (0.8%)
dizziness	1 (1.4%)	0
diarrhoea	1 (1.4%)	0
dehydration	1 (1.4%)	0
malnutrition	1 (1.4%)	0
rash	0	121 (100%)

In terms of characteristics of the lesions, documentation was poor, with 30% (36 of 121) of case-notes of patients with verruga peruana containing no information on the nature of the lesions. In those with better documentation, 13.2% (16) had lesions on the face, 48.8% (59) had lesions on the upper limbs and 48.8% (59) had lesions on the lower limbs.

Out of all chronic cases (n = 121), 14.9% (17) had a documented history of the acute febrile form of the disease. The majority, 82.4% (14), had this illness more than 28 days before the onset of the rash; three patients reported a febrile illness within the preceding 7 days.

Despite the high number of patients in which fever was documented in the history (80% of acute cases and 5% of chronic cases), only 22.5% (14 of 62) patients had a temperature of 37.5°C and above at the time of presentation.

### Diagnosis

For diagnosis, 84.8% (n = 162) of all patients had a blood smear examined, 4.7% (9) had a blood culture taken, and 15.2% (29) had no diagnostic testing performed, 26 of whom had chronic disease (Table 2).

**Table 2:** Results of diagnostic methods in acute and chronic patients.

Diagnostic method	Acute cases		Chronic cases	
Test result	Positive	Negative	Positive	Negative
Blood Smear	31/68 (45.6%)	37/68 (54.4%)	3/95 (3.2%)	92/95 (96.8%)
Blood Culture	8^1^	0	1^1^	0

^1^ only positive culture results were reported so the denominator (number of cultures taken) is not known

Of those with positive cultures, only two had positive smears and these were both patients with acute disease.

### Treatment

**Fig 5:**
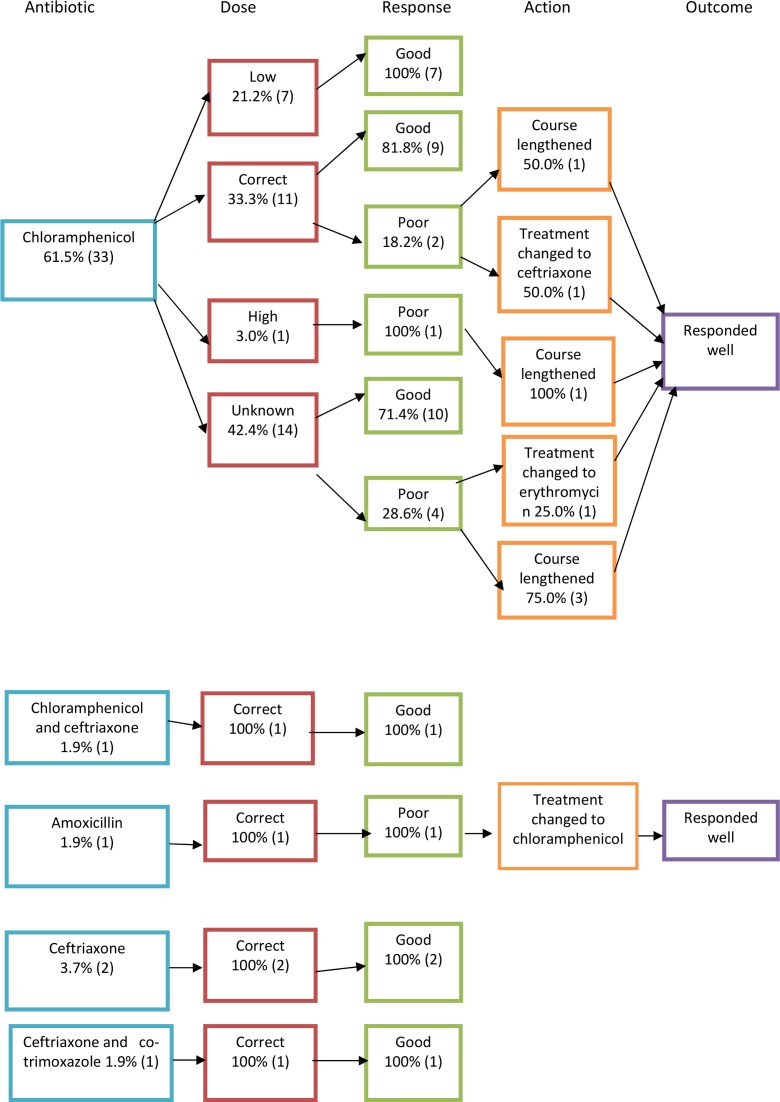
Treatment flowcharts for acute disease.

**Fig 6:**
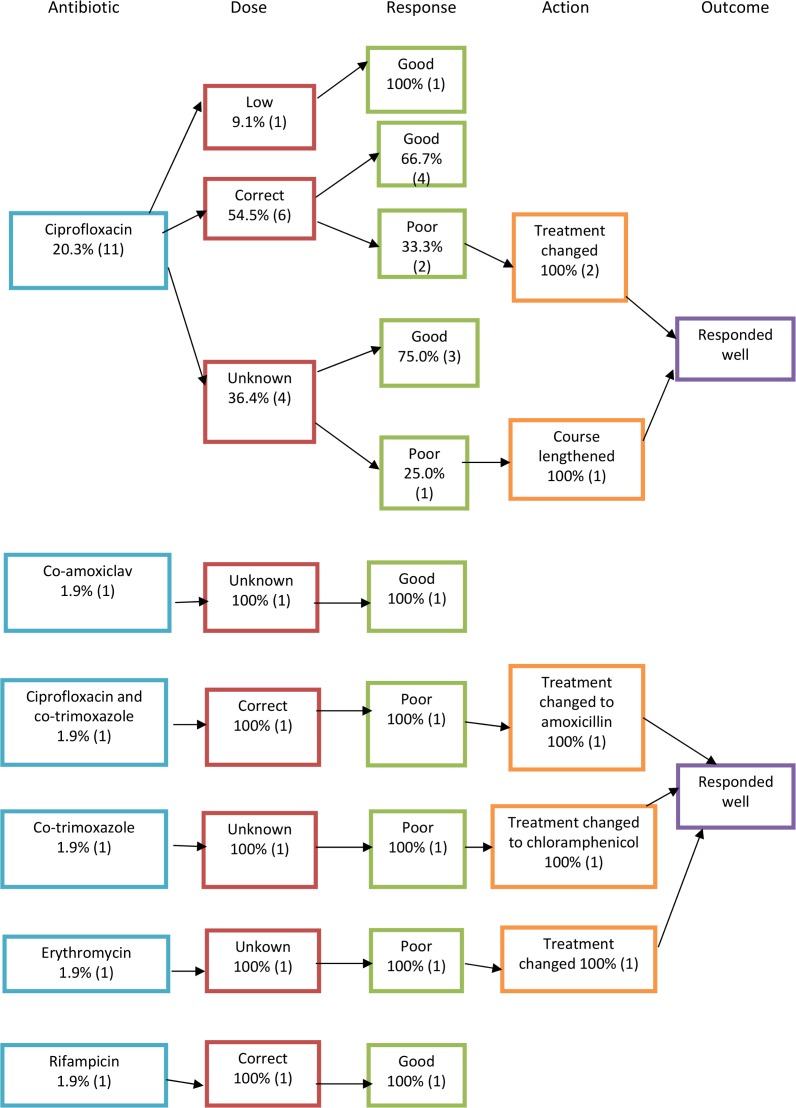
Treatment flowcharts for acute disease II.

**Fig 7:**
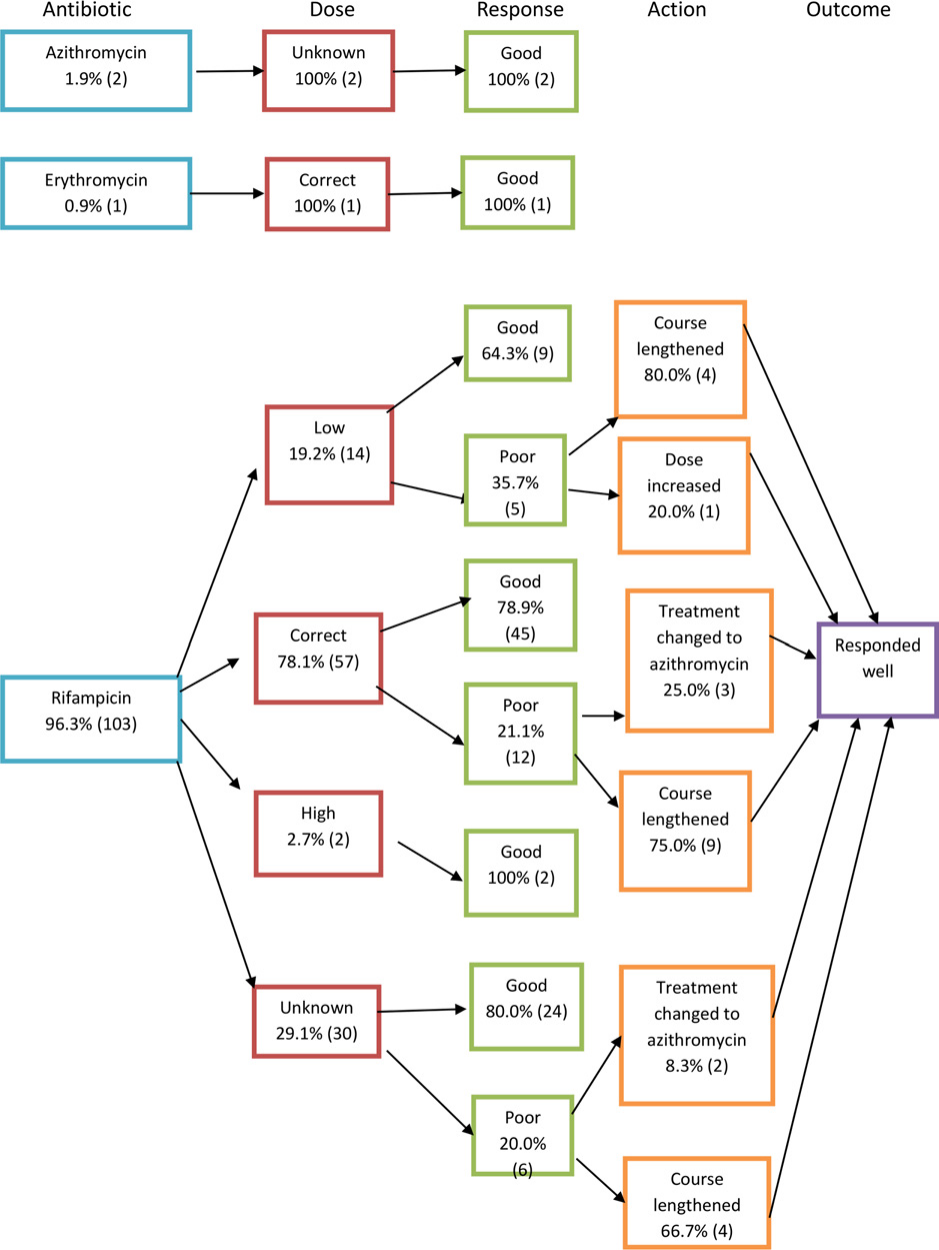
Treatment flowcharts for chronic disease.

In 54 out of 70 acute cases, the antibiotic given was clearly recorded in the case notes. In chronic cases, this was recorded in 106 out of 121 cases. Treatment flowcharts can be seen in Figs 5–7, which are summarised in Tables 3 and 4. To calculate percentages, the denominator used was the total number of cases where the antibiotic regime was recorded in the notes (54 in the case of acute and 106 in the case of chronic cases). Poor responders, as defined by the ministry of health guidelines [17], were those who returned to the healthcare facility with ongoing symptoms or who were inpatients who did not respond adequately to initial management who had ongoing positive blood smear or cultures for bartonellosis. Those that were presumed to have a ‘good response’ were those that were discharged with treatment and did not return to the health facility or inpatients that defervesced on the treatment given. Ministry of health treatment guidelines for the treatment of acute and chronic bartonellosis according to age and disease severity [17] have been translated into English and included in S1 File. The 1998 guidelines were the last to be published before the study period and were used to determine whether patients had been correctly dosed or over- or under-dosed (see Figs 5 and 6). Where dosing was not recorded in the notes, this is denoted in the treatment flowcharts as ‘unknown’.

**Table 3:** Acute Bartonellosis treatment regimes and outcomes.

Antibiotic regime	No. of cases treated with regime (n = 54)	Correct dose given	Total cases that responded well to initial dosing
Chloramphenicol	33 (61.1%)	11 (33.3%)	25 (75.8%)
Ciprofloxacin	11 (20.3%)	6 (54.5%)	8 (72.7%)
Ceftriaxone	2 (3.7%)	2 (100%)	2 (100%)
Chloramphenicol and Ceftriaxone	1 (1.9%)	1 (100%)	1 (100%)
Amoxicillin	1 (1.9%)	1 (100%)	0
Co-amoxiclav	1 (1.9%)	Unknown	1 (100%)
Co-trimoxazole	1 (1.9%)	Unknown	0
Ciprofloxacin and co-trimoxazole	1 (1.9%)	1 (100%)	0
Ceftriaxone and co-trimoxazole	1 (1.9%)	1 (100%)	1 (100%)
Erythromycin	1 (1.9%)	Unknown	0
Rifampicin	1 (1.9%)	1 (100%)	1 (100%)

**Table 4:** Chronic Bartonellosis treatment regimes and outcomes.

Antibiotic regime	No. of cases treated with regime (n = 106)	Correct dose given	Total cases that responded well to initial dosing
Rifampicin	103 (97.2%)	57 (55.3%)	80 (77.7%)
Azithromycin	2 (1.9%)	Unknown	2 (100%)
Erythromycin	1 (0.9%)	1 (100%)	1 (100%)

Chronic patients were mostly treated with rifampicin (96.3%, 103). Of these, 22.3% (23) had a poor response. This was attributed to under-dosing in 21.7% (5) of cases. The majority (73.9%, 17) of these poor-responders improved with a lengthening of their course of rifampicin but 21.7% (5) were switched to azithromycin with good therapeutic response.

Acute patients were mainly treated with chloramphenicol (63.5%, 33). Just over a fifth (21.2%, 7) responded poorly but this did not seem to be dose-related. The second most used antibiotic in the acute phase was ciprofloxacin, which was used in 20% (11) of cases. Ceftriaxone was used, alone or in combination, in 7.5% (4) of cases. Of those treated with ciprofloxacin alone, 27% (3 of 11) responded less than satisfactorily to the treatment. Two of these needed a change in treatment and the other responded well to an additional week of treatment.

Complications arose in 6.8% (13) patients. Out of these, 53.8% (7) had severe anaemia requiring multiple blood transfusions, 30.7% (4) had super-infections with salmonella, one had acute hepatitis and one had multiple adverse drug reactions.

### Pregnancy

There were two pregnant women in the sample, both had chronic disease and were treated with rifampicin. They made an uneventful recovery.

### Outcome

Most cases were treated as outpatients, and only 7.9% (15) were admitted to hospital. There were no deaths. It is noteworthy that ten patients presented having had multiple episodes of the illness in the preceding two years. One patient presented with his fifth episode of bartonellosis and another patient had suffered six episodes in the last two years.

## Discussion

These routine hospital-based data, from an outbreak of bartonellosis in an endemic area, confirm the findings of previous studies demonstrating that the burden of disease primarily falls upon children, with those aged 14 and under making up 65% of the cases in the sample studied.

There were more chronic cases (121) than acute (70) and chronic episodes occurred more frequently in younger people whereas in the older groups, acute and chronic disease occurred with similar frequency. This may reflect patterns of health-seeking behaviour for adults and for parents on behalf of their children; moreover without age group denominator it is not possible to make assumptions about age-specific attack rates.

Looking at seasonality, it is interesting to note that there are higher numbers of acute cases from March to May, just after the rainy season, when there are usually the highest numbers of vectors, and a reduction to low levels after. Whereas for chronic cases, there is a less marked peak from May to October after the acute phase peak. This fits with the findings reported in this study which show that in those reporting an eruptive disease following an acute episode, the majority have this over 4 weeks after the acute illness.

The clinical features that patients presented with are similar to those reported in previous studies. However, it is interesting to note that although 32.5% (62) of all patients reported fever, only 22.5% (14) were febrile at the time of presentation. This is consistent with the nature of fever in bartonellosis, which is known to wax and wane. This could be a possible explanation for the low positivity of blood smears among acute patients (45.6% in this study). It remains to be investigated whether taking blood smears during a febrile episode increases the yield of *B*. *bacilliformis*. In any case, it is important to remember that patients in this study were diagnosed clinically and treated empirically so it is highly probable that a proportion of cases had an alternative cause for fever that was not bartonellosis.

Around 50% of the patients came from surrounding villages outside Caraz, despite the fact that these areas all have their own health centres that offer free healthcare. One reason for this may be that these villages tend to have more cases and these spill over; another explanation could be the fact that during this time, bartonellosis research projects were taking place in Caraz Hospital and researchers were offering free rapid consultations, diagnostics and treatment in return for their participation in the study.

Blood cultures are not routinely taken in Caraz Hospital in patients with suspected bartonellosis but they were available during the research project. For logistical reasons, only positive cultures were recorded in the case notes, which explains why negative results were not noted during our data collection. Although still carried out in the majority (78.5%) of cases in Caraz Hospital, the positivity of blood smears during chronic disease is extremely low (3.2% or 3 of 95). From the cohort of acute patients that had both culture and smear taken, it was possible to calculate the crude sensitivity of blood smear for diagnosis of blood culture proven disease as a surprisingly low 25% (2 of 8). Current practice is to carry out a blood smear on every patient regardless of whether they present with acute or chronic disease. However, with a mere 3% of chronic cases having a positive smear, it raises the question of whether it is cost-effective to carry these out on chronic patients at all. Patients are treated empirically in endemic areas so the result of the blood smear does not alter the management.

In terms of management, the existent treatment guidelines were those published by the Ministry of Health in 1998 containing three different treatment options; for acute, non-complicated disease chloramphenicol was advised; for acute complicated disease, Penicillin G was added but could be substituted with a quinolone if no improvement was seen after 72 hours and dexamethasone was added in the event of neurological compromise; and for chronic disease streptomycin or rifampicin were advised. However during the period covered by this investigation a study comparing azithromycin to rifampicin was being conducted in Caraz.^18^ Responses to azithromycin appear to have been favourable, in contrast to treatment with rifampicin where 22.3% (23) showed a poor response. Azithromycin has been in fact been replaced as first line treatment for chronic disease in the latest Ministry of Health guidelines published in 2006. Similarly, in acute patients treated with chloramphenicol, 21.2% (7) responded poorly initially but the majority (71%, 5) responded well after an extended period of treatment. It was interesting to note that although guidelines suggest that for acute severe cases, Penicillin G should be added, none of the severe cases had this prescribed.

It is of interest to look at the cases treated with ciprofloxacin due to the recent negative attention from *in vitro* studies [18, 19]. Eleven cases were treated with ciprofloxacin alone and 27% (3) had a poor response. One of these cases was severely unwell and developed hepatitis; he required a switch to ceftriaxone. The second case was switched to chloramphenicol and the third responded well to an extra few days of treatment. Thus, the majority of patients responded well to the antibiotic and, in fact, the poor response rate was comparable to that of the other treatments for both acute and chronic disease. This is *in vivo* evidence that resistance problems may not be as marked as suggested by recent studies [18, 19]. However, more *in vivo* research needs to be done in this area before a firm conclusion can be reached.

Finally, many patients had to return to hospital to receive an extra week of antibiotics, particularly in the chronic phase, so it may be of interest to study more closely whether antibiotic courses should be routinely lengthened, particularly if this renders the patient abacteraemic, as this would help in reducing transmission. In the latest Ministry of Health guidelines, the course of rifampicin (which is now 2^nd^ line for chronic disease) has been lengthened from 14 to up to 28 days.

Outcomes were very good in this cohort with no deaths or serious complications and an admission rate of just 7.9%. The two pregnant women also had uneventful recoveries.

From an immunological point of view, it is intriguing to find that a proportion of patients, 5.23% (10) had repeated episodes in a short period of time. There could be many explanations for this; it could mean that illness episodes are only partially treated and so the illness recrudesces, or it could imply multiple infections in which case, this means that immunity following infection is incomplete in some people. The other explanation, recently highlighted in a study carried out in the same region of Peru, is the genetic variability of *Bartonella*. In fact, two novel species (*B*. *rochalimae* and *Candidatus* B. ancashi) have recently been identified [20], the latter of which was found to cause a mild version of the chronic eruptive form without many systemic features. There may be many other species which await discovery and since the immunological aspects of this disease have not been well studied, there remain many unanswered questions.

Due to time constraints, it was not possible to visit a sample of smaller health centres around Caraz to obtain case notes. Therefore, the sample may not be representative of the Huaylas province as a whole and may be biased towards more severe cases as these may be the ones who have travelled some distance to the hospital to be treated. It also means that it was not possible to compare how patients are treated in terms of diagnostic tests and management between hospital and health care post settings. It was also only possible to look at a sample of cases from one year of a long outbreak period (2002–2008) and it may be that this is not representative of the entire time period.

Documentation in the case notes was quite poor, therefore limited amount of detailed information was able to be obtained, particularly in chronic cases where details of the nature and number of lesions were often absent. This is one of the limitations of a retrospective study design.

This study was carried out in 2010 and used data from the peak year of the last outbreak (2003). According to surveillance data collected and published by the Ancash health board [21], the number of cases of bartonellosis have remained stable in the region in the last four years. Between 2012 and 2015 thus far, there have only been two occasions where the number of cases have exceeded the alert level. There was a peak in chronic cases in week 3 of 2012 and a peak of acute cases in week 25 of 2014. Otherwise, the number of cases have remained in the stable or safe zone indicating that there has not been a further outbreak.

In conclusion, diagnostic methods for acute and chronic *Bartonella bacilliformis* remain unsatisfactory, with those that are cheap and available, such as blood films and cultures, having a low sensitivity and those that have a potentially higher sensitivity, such as PCR, being more expensive and impractical in most health centres in endemic regions.

Randomised controlled trials of drug therapy for acute and chronic bartonellosis need to be carried out as the current guidelines are supported by very weak evidence, and such studies should be carried out in endemic areas as trials carried out in tertiary hospitals in large cities may not be generalisable to the population of interest. In addition, development of effective surveillance tools to inform understanding of the epidemiology of disease and readiness for outbreak investigation will all be important in the control of this ancient and neglected tropical disease.

## Supporting Information

S1 File Ministry of health 1998 treatment guidelines for the management of acute and chronic bartonellosis. (DOCX)

S1 Text Questionnaire used for retrospective study. (DOCX)
