# Supplementary material for: An Outbreak of Bartonella bacilliformis in an Endemic Andean Community
Source: PLoS One. 2016 Mar 18;11(3):e0150525. doi: 10.1371/journal.pone.0150525 (PMC4798250; doi:10.1371/journal.pone.0150525)
Supplement: S1 File — (DOCX) [file pone.0150525.s001.docx]

Ministry of health 1998 treatment guidelines for the management of acute and chronic bartonellosis

| Disease type | Drug | Scheme |
| --- | --- | --- |
| Acute, uncomplicated | Chloramphenicol | 50mg/kg/day for 3 days in 3 divided doses (IV or PO) then 25mg/kg/day PO to complete 14 days |
| Acute, severe/complicated | Chloramphenicol  And  Penicillin G | 50mg/kg/day TDS (IV) for 14 days  2000 IU 4-hourly (IV) for 14 days.  (If no clinical improvement after 72 hours, replace Penicillin G with a quinolone and consider blood transfusion) |
| With neurological compromise (somnolence, stupor, coma) | Add Dexamethasone | 4mg TDS IV for the first 72 hours of antibiotic treatment |
| Chronic, adults and adolescents ≥ 15 years | Rifampicin | 600mg OD PO for 14 days. |
| Chronic, children <15 years | Rifampicin | 10mg/kg/day OD PO for 14 days.  (If no clinical improvement, give Streptomycin 15mg/kg/day (max. 750mg/day) IM for 10 days) |
