# Supplementary material for: An Outbreak of Bartonella bacilliformis in an Endemic Andean Community
Source: PLoS One. 2016 Mar 18;11(3):e0150525. doi: 10.1371/journal.pone.0150525 (PMC4798250; doi:10.1371/journal.pone.0150525)
Supplement: S1 Text — (DOCX) [file pone.0150525.s002.docx]

Questionnaire used for retrospective study

**General information**

Patient code: Date presented: Gender:

Age: Under 1/1-4/5-9/10-14/15-19/20-39/40-60/over 60

Address of patient:

Other family members affected: Y/N Pregnant: Y/N If so, how many weeks?

**Vitals signs**

Pulse: BP: Temperature: Respiratory rate:

**Symptoms and signs**

Fever: Y/N Joint pain: Y/N Cough: Y/N Diarrhoea: Y/N Vomiting: Y/N

Anorexia: Y/N Jaundice : Y/N Headache: Y/N Malaise: Y/N Dizziness: Y/N

Anaemia: Y/N Dehydration: Y/N Malnutrition: Y/N

Rash: Y/N How many skin lesions? 1-5/6-10/11-15/16-20/>20

Where? Face: Y/N Trunk: Y/N Upper limbs: Y/N Lower limbs: Y/N

Did they have a febrile illness before the rash? If so, how long before? <7 days/7-14/15-21/22-28/>28

**Diagnosis**

Diagnostic test 1: Blood smear/culture/PCR Positive?: Y/N

Diagnostic test 2: Blood smear/culture/PCR Positive?: Y/N

**Treatment**

Treatment 1: Course length: <7days/7-14/15-21/22-28/29-35/>35 Dose: As per guidelines/less/more

Treatment 2: Course length: <7days/7-14/15-21/22-28/29-35/>35 Dose: As per guidelines/less/more

Treatment 3: Course length: <7days/7-14/15-21/22-28/29-35/>35 Dose: As per guidelines/less/more

**Complications**

None: Y/N Salmonella super-infection: Y/N Other super-infection: Y/N Which?

Renal: Y/N Neurobartonellosis: Y/N Respiratory: Y/N Hepatic: Y/N

Obstetric: Y/N Gastrintestinal: Y/N Haemodynamic: Y/N Oedema: Y/N

Other: Y/N Which?

**Outcome**

Admitted?: Y/N Time to recovery: <3 days/3-7/8-14/15-21/22-28/29-35/36-42

Devloped verruga after acute disease?: Y/N How long after? <1 week/1-2/3-4/>4weeks

Re-presented to hospital?: Y/N How many times?: Once/twice/3 times/>3 times

Outcome: Treatment changed/treatment lengthened/dose changed/no change

Died?: Y/N
